# Mechanism for Nucleotidyl Transfer in LINE-1 ORF2p Revealed by QM/MM Simulations

**DOI:** 10.3390/ijms26178661

**Published:** 2025-09-05

**Authors:** Igor V. Polyakov, Kirill D. Miroshnichenko, Tatiana I. Mulashkina, Anna M. Kulakova, Maria G. Khrenova

**Affiliations:** 1 Chemistry Department, Lomonosov Moscow State University, 119991 Moscow, Russia; polyakoviv@gmail.com (I.V.P.); kirmir145@gmail.com (K.D.M.);; 2 Institute of Biomedical Chemistry, 119121 Moscow, Russia

**Keywords:** ORF2p, reverse transcriptase activity, DNA elongation, QM/MM MD, Mg^2+^ cation

## Abstract

The Long Interspersed Element-1 (L1) retrotransposon is an ancient genetic parasite that comprises a significant part of the human genome. ORF2p is a multifunctional enzyme with endonuclease (EN) and reverse transcriptase (RT) activities that mediate target-primed reverse transcription of RNA into DNA. Structural studies of LINE-1 ORF2p consistently show a single Mg^2+^ cation in the reverse transcriptase active site, conflicting with the common DNA polymerase mechanism which involves two divalent cations. We explored a reaction pathway of the DNA elongation based on the recent high-resolution ternary complex structure of the ORF2p. The combined quantum and molecular mechanics approach at the QM (PBE0-D3/6-31G**)/MM (CHARMM) level is employed for biased umbrella sampling molecular dynamics simulations followed by umbrella integration utilized to obtain the free energy profile. The nucleotidyl transfer reaction proceeds in a single step with a free energy barrier of 15.1 ± 0.8 kcal/mol, and 7.8 ± 1.2 kcal/mol product stabilization relative to reagents. Concerted nucleophilic attack by DNA O3′ and proton transfer to Asp703 occur without a second catalytic metal ion. Estimated rate constant ∼60 s^−1^ aligns with RT kinetics, while analysis of the Laplacian of the electron density along the cleaving P-O bond identifies a dissociative mechanism.

## 1. Introduction

The Long Interspersed Element-1 (LINE-1, L1) retrotransposon is an ancient genetic parasite that comprises a significant part of the human genome. Studies in the pathophysiology of cancer, autoimmunity, and ageing [1,2,3,4] implicate LINE-1 involvement. The L1 “copy-and-paste” mechanism is driven by the ORF (open reading frame) proteins [5]. The ORF1p is a nucleic acid chaperone and RNA packaging protein which consist of three domains: N-terminal coiled-coil, RNA-recognition motif (RRM) and C-terminal (CTD) [6]. The ORF1p assembles into trimers via an N-terminal coiled-coil stabilized by metal ions, thus forming a scaffold for RNA binding. ORF2p is a multifunctional enzyme with endonuclease (EN) and reverse transcriptase (RT) activities that mediates target-primed reverse transcription of RNA into DNA [7]. The ORF2p consists of endonuclease (EN) domain (1–238), reverse transcriptase (RT) core (238–1061), and the C-terminal (CTD) domain [8,9]. Structural and evolutionary analysis confirms that ORF2p shares the canonical polymerase fold found in other RNA- and DNA-dependent polymerases [8,9]: the tower (238–440), fingers (440–558), palm (558–776), thumb (776–882), and wrist (882–1061) domains comprise the RT core. Structural studies [8,9,10] have recently revealed the active site architecture of ORF2p: for example, the 2.1 Å crystal structure of the ORF2p RT core in a ternary complex (PDB ID: 8C8J [9]) shows RNA_12_–DNA_9_ template–primer heteroduplex and an incoming thymidine triphosphate (dTTP) bound in a right-hand polymerase fold. The palm, fingers, and thumb domains form a moiety that cradles the RNA template–DNA primer heteroduplex. Authors note [9] that the overall configuration of the active site is highly conserved throughout RTs [11,12] and related polymerases which should result in a similar catalytic mechanism. Three invariant aspartate residues—Asp600, Asp702, and Asp703—reside in the active site near the Mg^2+^ ion which positions the incoming nucleotide (dNTP). The high-resolution structure PDB ID: 8C8J [9] demonstrates that Asp600 and Asp702 side chains are involved in the Mg^2+^ coordination shell while the Asp703 side chain is closer to the 3′ end of the DNA primer. The aromatic side chain of the Phe605 residue is stacked to the sugar ring of the dNTP which sterically excludes ribonucleotides (with 2′-OH group), explaining why ORF2p cannot function as an RNA-dependent RNA polymerase. A recent computational study [13] was focused on overall structure and dynamics of the ORF2p without specific consideration for the reaction mechanism or the active site structure and dynamics.

Next, we will discuss in detail experimental and computational data on reaction mechanisms in active sites of related enzymes, polymerases, and reverse transcriptase to clarify what is already solid knowledge and what should be additionally verified.

Nucleic acid polymerases generally employ the two-metal-ion mechanism [14,15] that was originally proposed by Thomas Steitz [16,17] based on the two-metal-ion enzymatic mechanism for the 3′,5′-exonuclease reaction of *Escherichia coli* DNA polymerase I [16,18,19]. This two-metal-ion catalysis is now considered universal among polymerases [20], where one divalent cation (Mg^2+^ or similar) coordinates the triphosphate of dNTP, while the other divalent cation assists deprotonation of the primer 3′-OH. After the initial deprotonation step, the phosphoryl transfer occurs as follows: the primer 3′ oxygen atom attacks the α-phosphate of the dNTP, forming a new O-P bond while breaking the bridging O-P bond between α- and β-phosphate groups, releasing inorganic pyrophosphate (PPi). The negative charge on the leaving pyrophosphate group is stabilized by one of the divalent cations.

Experimental studies of DNA polymerases β, η, and λ revealed the binding of the third divalent cation [21,22,23] between the α- and β-phosphates of dNTP without coordination of other active site residues. It is not clear whether the role of the third cation is “catalytic” or “product-stabilizing”, i.e., if the binding occurs before or after the chemical step of the reaction.

Details of the reaction mechanisms of nucleotide polymerases were uncovered through structural, mutational, and molecular modeling studies in the past years [24,25,26,27]. The combined quantum and molecular mechanics (QM/MM) approach [28] was utilized to study the reaction mechanisms of nucleotide polymerases and, specifically, RTs. Earlier studies [29,30,31,32,33,34] considered the two-metal mechanism in different polymerases. Simulations were carried out for both the nucleophile formation accompanied by the proton transfer and phosphoryl transfer steps, while the latter was found to be the limiting in most of the studies. Amino acid residues carrying negatively charged carboxylates in the active site can act as proton acceptors in the first step. Alternatively, a proton can be transferred to the oxygen atom of phosphate via the water-mediated and substrate-assisted (WMSA) mechanism. Depending on the particular study, different proton acceptors were suggested, as follows: a side chain of Asp256 residue; [29] oxygen atom of the α-phosphate of DNA polymerase β through WMSA; [30] and a side chain of Asp490 of DNA polymerase λ; [31] WMSA for T7 DNA Polymerase (not the Asp654) [33]. The limiting P-O bond formation step was estimated at around 28 kcal/mol for DNA polymerase β; [30] 17 kcal/mol for DNA polymerase λ; [31] 15 (free energy) to 25 (potential energy) kcal/mol for T7 DNA Polymerase; [33] and 20–30 kcal/mol depending on the starting structure for DNA polymerase IV (Dpo4) [32]. Importantly, the QM/MM products are found to be higher compared to the reactants in all these studies.

The more recent studies considered HIV RT [35], RNA-dependent RNA polymerase (RdRp) from SARS-CoV-2 [36] and DNA polymerase κ [37]. For both RT and RdRp the two-metal-ion mechanism was considered, while three metal cations were considered for DNA polymerase κ. HIV RT reaction barriers were found to be 14–16 kcal/mol while the reaction products were 10 kcal/mol higher than reactants. The barrier of the P-O bond formation step was determined as 15 kcal/mol in the RdRp system, while proton transfer required only 8 kcal/mol [36]. WT and cancer-related mutated Y432S systems were simulated for DNA polymerase κ [37]. It was found that the Y432S mutation induces structural effects on the active site, altering the coordination mode of Mg^2+^ (1) and Mg^2+^ (3) metal cations, and also results in an increase in the energy barrier from 9.3 to 13.9 kcal/mol and 6 kcal/mol destabilization of the reaction products relative to reagents compared with the 2.2 kcal/mol stabilization in the WT enzyme. Remarkably, QM/MM results in these studies are focused on the energy landscape of the P-O bonds cleavage and formation, but not the proton transfer step. In ref. [36], authors suggest that the proton from the 3′-OH can be transferred to the PPi formed on the previous step and thus be prepared for the next elongation reaction. Importantly, this step is highly unfavorable; it happens with a relatively low energy barrier of 8 kcal/mol, but the state with the deprotonated O3′ and protonated PPi is ~7 kcal/mol higher than the initial state with the neutral 3′-OH and fully deprotonated PPi. In ref. [35], authors explicitly state that they removed a hydrogen from the O3′ of the guanine base, preparing it for the phosphoryl transfer reaction. Authors of ref. [37] do not perform calculations for the proton transfer process and refer to the literature data, stating that this is not a limiting step. At that, we should conclude that among recent computational studies that are performed at reliable theory levels, there are none that succeed in explicit determination of the proton acceptor during the enzymatic reaction. Moreover, a comprehensive experimental study, which includes kinetic isotope effect determination, states that the limiting step involves proton transfer for a set of examined RNA- and DNA- dependent RNA and DNA polymerases [38]. All these discrepancies raise the following questions: Are the proton transfer and P-O bonds formation and cleavage stepwise or concerted processes? Do the modeled active site structures represent the reactive conformation, and is Mg^2+^ coordination of the O3′ atom obligatory for catalysis to proceed?

We analyzed the mechanistic data on other enzymatic reactions of P-O bond formation and cleavage occurring with guanosine and adenosine triphosphates (GTP and ATP) [39,40,41,42,43]. The recently computed energy profile of the GTP hydrolysis by RAS-GAP complex [44] confirms earlier findings [45,46], that reasonable reaction barriers are obtained for cleavage and formation of P-O bonds in triphosphate with only one divalent cation (Mg^2+^) in the active site. This cation coordinates the phosphates and is not involved in the activation of the attacking water oxygen atom. The same is true for the ATP nucleotide in the myosin motor protein [47]. The other case is the adenylate cyclase (AC) [48], where the 3′ sugar oxygen atom attacks the α-phosphate, then pyrophosphate is eliminated forming the cyclic adenosine monophosphate. The active site of AC includes not one, but two Mg^2+^ ions [49]. Recent computer simulations [49,50] disagree on roles of active site cations. While in ref. [49] both magnesium cations coordinated ATP phosphate groups and aspartate side chains, the later study presumed that the O3′ atom was involved in one of the Mg^2+^ coordination spheres [50]. The reaction was energetically favorable and yielded a 15 kcal/mol energy barrier in the former case, while in the latter case, the barrier was computed as 20 kcal/mol and rection products were higher in energy than reactants by ~18 kcal/mol. This discrepancy makes it unclear whether the second magnesium cation should activate the attacking 3′ oxygen atom or if its role is limited to coordination and positioning of the triphosphate tail of ATP. The P-O bond cleavage is discussed in detail in the recent computational study [51] of mechanisms of nucleophilic substitution at phosphorus centers of the organophosphates in 15 enzymes. More often, Mg^2+^ cations are involved in the proper organization of the active site and polarize P-O bonds of a reactive phosphate group to obtain the electrophilic site on the phosphorus atom.

Despite the existence of generally accepted two-metal-ion catalytic mechanisms in RTs and polymerases, not all issues are completely clarified. The X-ray data is also controversial and does not derive a consistent conclusion on the number of Mg^2+^ cations in the active site. The widely used crystal structure of the HIV-1 RT PDB ID: 1RTD [52] carries two Mg^2+^ cations and it is usually utilized as a source of coordinates in computer simulations. This structure traps reagent-like complex, but the reaction does not proceed as the primer does not contain the catalytic 3′-OH group. One of two Mg^2+^ cations in this structure has a complete coordination sphere composed of six oxygens, plays a structural role, and polarizes the P-O bond of the α-phosphate group. The second Mg^2+^ has only one resolved coordination bond with the oxygen atom of the Asp185 that seems quite untypical for Mg^2+^ containing biomolecular crystals. Other structures, including PDB ID: 7SR6 [12], 1R0A [53], and 5TXM [54], contain only one Mg^2+^ cation with the complete coordination sphere composed of six ligands similarly to the first Mg^2+^ discussed for the PDB ID: 1RTD [52] structure. Similarly, a single cation active site is observed in the recent structure of the ORF2p reverse transcriptase PDB ID: 9HDP [10]. This raises the question of probing the alternative computational models exploring reaction pathways when only one “structuring” Mg^2+^ is present in the active site.

Herein, we perform QM/MM molecular dynamic simulations of the reaction mechanism of the DNA chain elongation in the active site of the RT domain of the ORF2p. We aim to clarify the following issues: How the α-β phosphate bond of an incoming nucleoside triphosphate is cleaved and how the nucleoside is added to the growing nucleic acid chain. What is the proton acceptor in this reaction? Does the limiting step of chemical transformation include the proton transfer? To our best knowledge, no simulations of the ORF2p reaction mechanism are currently available in the literature. Given the presence of only one magnesium cation in the active site in the crystal structure of the ORF2p, we explore the feasibility of a one-metal mechanism in this specific enzyme case.

## 2. Results and Discussion

**Figure 1:**
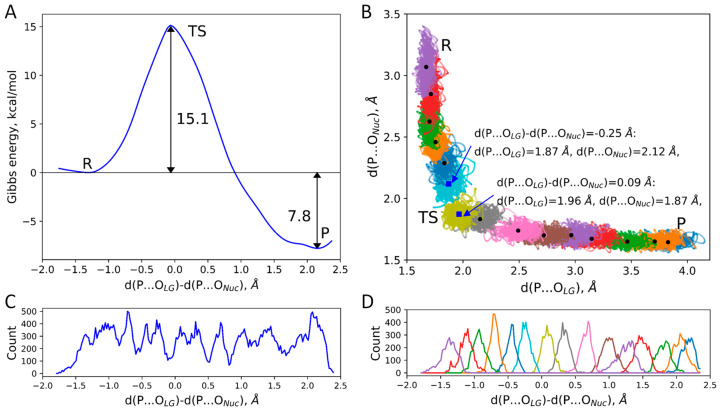
(**A**) Gibbs energy profile of the dTTP nucleotide addition to the single-strand DNA in the ORF2p active site. (**B**) Distributions of d(P…O_LG_) and d(P…O_Nuc_) in umbrella sampling runs with different biasing potentials (colored dots). Large black dots correspond to the mean values of these two coordinates in each window. Large blue squares are mean values in windows that are closest to the TS. Reagents, transition state, and products regions are marked as R, TS, and P, respectively. (**C**) Cumulative curve of all data points accumulated from all MD trajectories with biasing potentials added to collective variable. (**D**) Distributions of reaction coordinate obtained in umbrella sampling simulations. Colors match “windows” on panels (**B**,**D**). For both (**C**,**D**), plot curves represent histograms with the reaction coordinate values from −2.5 to 2 Å divided into 200 equal bins.

The Gibbs energy profile of the DNA polymerization reaction in the active site of ORF2p was obtained at the QM/MM MD level utilizing umbrella sampling simulations (Figure 1A). Reaction proceeds via a single elementary step with the energy barrier of 15.1 ± 0.8 kcal/mol, that corresponds to the rate constant of around 60 s^−1^ at 300 K according to the transition state theory. The error coming from the statistical analysis was 0.8 kcal/mol that corresponds to the range of rate constants from 12 to 300 s^−1^. Reaction products were stabilized relative to reagents by 7.8 ± 1.2 kcal/mol. This is a meaningful result as it promotes the following steps of polymerization and elongation of the DNA chain. There are no experimental data on the kinetics of this reaction in ORF2p; however, we can compare this value with the RT enzymes. The eubacterial RT MarathonRT (MRT) operates with the a rate constant of 25 s^−1^ [55]. The rate constant of dNTP incorporation by HIV-1 RT ranges from 0.1 to 35 s^−1^ depending on different factors [56]. Thus, we can conclude that we obtained reasonable results.

**Figure 2:**
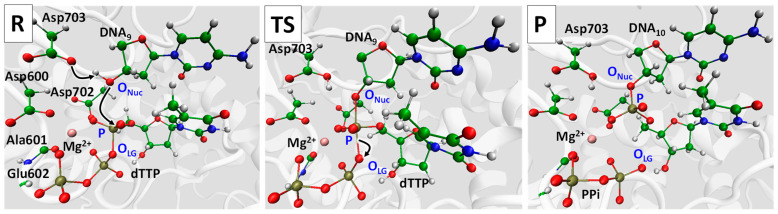
Mechanism of the DNA elongation in the RT domain of the ORF2p. Snapshots correspond to stationary point regions: R—reagents, TS—transition state, P—products. dTTP—thymidine triphosphate, PPi—pyrophosphate. Color code: carbon—green, oxygen—red, nitrogen—blue, and phosphorus—ochre, magnesium—pink, and hydrogen—white.

Next, we will discuss in detail the reaction mechanism (Figure 2). This single step reaction was initiated by a nucleophilic attack of the phosphorus atom of the α-phosphate group of the dTTP by the O3′ atom of the DNA hydroxyl group denoted here as O_Nuc_. This process was accompanied by the proton transfer from the O3′ atom to the oxygen atom of the side chain of Asp703. As a reaction product, the DNA was elongated (DNA_9_ → DNA_10_) and pyrophosphate was formed.

We plotted distributions of cleaving and forming bonds from MD trajectories of all umbrella sampling runs (Figure 1B). We analyzed reaction coordinate values and individual interatomic distances, d(P…O_Nuc_) and d(P…O_LG_), for two biased trajectories that were closest to the transitions state region corresponding to the reaction coordinate of −0.06 Å (Figure 1A). Next, we extracted only MD frames from these two runs with reaction coordinate values close to the TS, from −0.08 Å to −0.04 Å to evaluate the mean cleaving and forming bond lengths at TS. The distance of the nucleophilic attack was about 2 Å and the cleaving bond length was ~1.9 Å. According to the Pauling’s equation of determination of the mechanism type from the interatomic distances at TS [57], the reaction occurred via dissociative mechanism with the probability being 0.57. This value is quite close to 0.5; therefore, we performed detailed analysis of the ES complex following a recent study [51].

**Figure 3:**
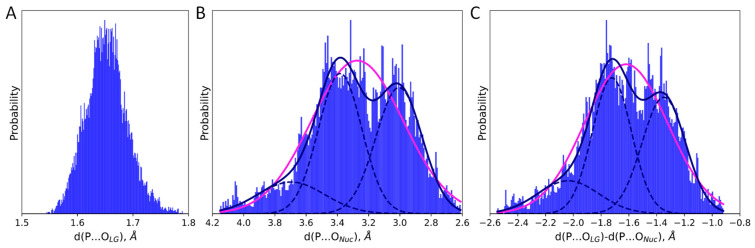
Distributions of distances in the unconstrained QM/MM molecular dynamics of the ES complex for the (**A**) d(P…O_LG_), (**B**) d(P…O_Nuc_), and (**C**) d(P…O_LG_)-d(P…O_Nuc_). The magenta line corresponds to the unimodal normal distribution and the navy solid line is a sum of three normal distributions (navy dashed lines for each component) with the corresponding weights.

Analysis of the QM/MM MD trajectory of the ES complex depicts high heterogeneity of states (Figure 3). The distribution of distance of the nucleophilic attack, d(P…O_Nuc_) is wide and can be described by a combination of at least three normal distributions (Table 1). We can discriminate two major fractions with almost equal weights corresponding to a tighter (d(P…O_Nuc_) = 3.01 ± 0.02 Å) and more relaxed (d(P…O_Nuc_) = 3.38 ± 0.02 Å) types of states. The distribution of the d(P…O_LG_) was unimodal and narrow (Figure 3A). If we compare distributions of d(P…O_Nuc_) (Figure 3B) and d(P…O_LG_)-d(P…O_Nuc_) (Figure 3C), we can find that their shapes are practically the same, being both characterized by a combination of three normal distributions with the same weights of corresponding fractions.

**Figure 4:**
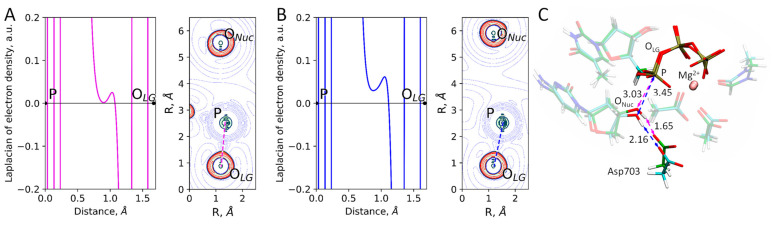
The Laplacian of the electron density calculated at stationary points, corresponding to (**A**) tighter (ES^T^) and (**B**) looser (ES^L^) ES complexes along the cleaving P-O_LG_ bond and in the plane of a phosphorus atom, P, and O_LG_, O_Nuc_ oxygen atoms. Contour lines are ± (2;4;8)·10^n^ a.u., −2 ≤ n ≤ 1, blue dashed contour lines indicate the electron density depletion areas (∇^2^ρ(**r**) > 0), red solid lines identify the electron density concentration (∇^2^ρ(**r**) < 0), and the green solid line corresponds to ∇^2^ρ(**r**) = 0. The area with ∇^2^ρ (**r**) < 0 is colored in light green. The P-O_LG_ bond is shown by a dashed line. (**C**) Alignment of the QM parts of the ES^T^ (carbon in green) and ES^L^ (carbon in cyan) models. Key differences are represented by interatomic distances (in Å).

The reaction coordinate at the minimum of the reagent states was −1.30 Å (Figure 1), which is close to the mean value of the normal distribution, corresponding to the tightest type of ES states (Table 1). Therefore, the equilibrium geometry configuration obtained from a QM/MM MD frame from this fraction should likely be representative to determine the reaction mechanism type. Still, we also obtained another minimum corresponding to the second major fraction of ES states. Thus, we obtained two ES complexes, a tighter one, ES^T^, with the distance of the nucleophilic attack being 3.03 Å, and a looser one, ES^L^, with d(P…O_Nuc_) = 3.45 Å (Figure 4C). The Laplacian of the electron density was calculated along the P-O_LG_ bond for both structures (Figure 4) as suggested in ref. [51] to reveal the type of the reaction mechanism. For both minima, there is no electron density concentration area on the P-O_LG_ bond line, which is an indication of the dissociative mechanism.

## 3. Materials and Methods

**Figure 5:**
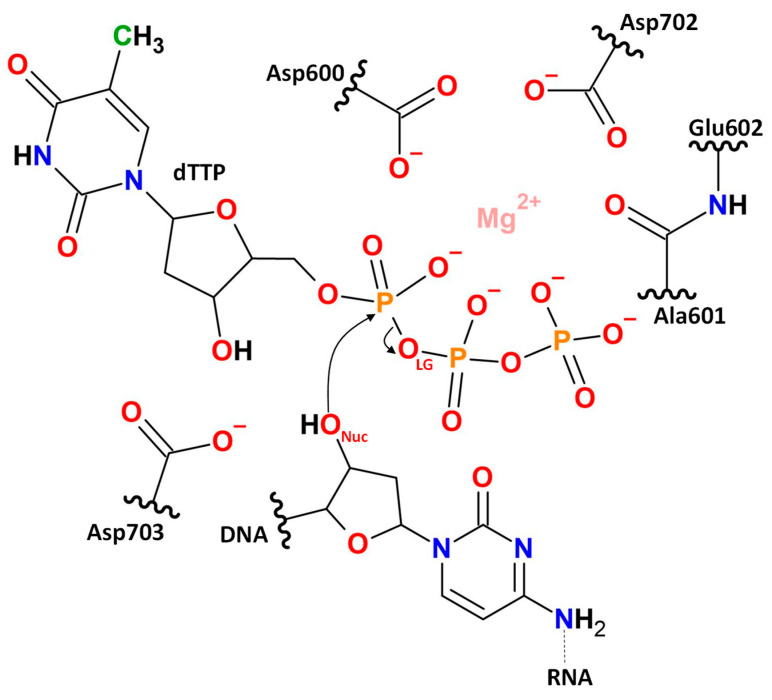
The QM subsystem includes the Mg^2+^ cation, side chains of Asp600, Asp702, and Asp703, amino acid residues, main chains of amino acid residues Ala601 and Glu602, the dTTP nucleotide, and a part of the nucleotide from DNA.

We utilized the model system obtained in the previous study [13] that was originated from the crystal structure PDB ID: 8C8J [9] that represented a triple complex of ORF2p with thymidine triphosphate nucleotide (dTTP) and an RNA_12_–DNA_9_ heteroduplex. The data of the 500 ns production run [13] was analyzed and representative structure was utilized as a starting point for QM/MM MD simulations. The QM subsystem (Figure 5) included a Mg^2+^ cation, side chains of the residues Asp600, Asp702, and Asp703, parts of the main chains of the amino acid residues Ala601 and Glu602, the dTTP nucleotide, and a part of DNA. The QM subsystem comprised 91 atoms with the −5 total charge in a singlet state. The QM subsystem was described at the Kohn–Sham DFT level with the hybrid functional PBE0 [58] with empirical dispersion correction D3 [59] and 6-31G** basis set. Selection of the QM level is always a compromise between accuracy and computational efforts as the QM system usually exceeds 100 atoms. There is already a clear understanding that the optimal theory level is a hybrid functional with dispersion correction with a double-zeta basis set with polarization functions [28,60]. This is in line with the recent DFT benchmark study of a chemical reaction dataset that demonstrates that addition of polarization functions in double-zeta basis sets considerably increases accuracy, whereas the effect of the extension to the triple-zeta basis is around 1 kcal/mol [61]. The MM subsystem was described using CHARMM36 [62,63,64] and TIP3P [65] force fields. The calculations were performed using software packages TeraChem v1.93P [66] and NAMD 2.14 [67] with the QM/MM interface [68]. All QM/MM MD simulations were performed in the NPT ensemble at 300 K and 1 atm with 1 fs integration time step. Pressure and temperature were maintained with the Nosé–Hoover Langevin piston pressure control [69] and the Langevin dynamics [70].

We started with the unconstrained QM/MM dynamics of the ES complex. It was performed for 25 ps and the last 15 ps were utilized for analysis. Next, QM/MM MD umbrella sampling simulations with the addition of biasing potentials to the reaction coordinate were performed to obtain a Gibbs energy profile of the polymerization reaction in the DNA active site of ORF2p. The reaction coordinate was selected as a difference of two distances d(P…O_LG_)-d(P…O_Nuc_), the distance between phosphorus and oxygen atoms of the leaving group, d(P…O_LG_), and between phosphorus and oxygen of the hydroxyl group at the 3′ end of the DNA, d(P…O_Nuc_) (Figure 1 and Figure 5). The reaction coordinate was divided into 15 windows and harmonic potentials were centered at the values from −1.7 to 2.6 Å. The force constants of the harmonic potentials in the transition state region were k = 80 or 120 kcal/(mol·Å^2^), and k = 40 kcal/(mol·Å^2^) was applied in regions close to the ES and products minima. The production run for each trajectory was 5 ps. Weighted histogram analysis (WHAM) [71,72] and umbrella integration (UI) [73] were utilized to reconstruct a Gibbs energy profile from statistical analysis of distributions of reaction coordinates in the umbrella sampling trajectories. The force constants were selected in such a way that distributions of reaction coordinates from neighboring “windows” overlap. Proper accumulation of statistics was monitored from reaction coordinate distributions in separate runs and their overlaps, as well as the overall coverage of the reaction coordinate (Figure 1C,D).

Frames from two major states (Figure 4) of the QM/MM MD simulation of the ES complex (Figure 3) were selected for the following QM/MM geometry optimization. These structures were used to locate minima on the potential energy surface, corresponding to different representative ES complexes. QM/MM optimization was performed using the Tcl ChemShell 3.7.1 software [74] with the efficient DL-FIND optimizer [75] and TURBOMOLE 7.6 quantum chemistry software package [76]. The quantum subsystem and theory level were selected the same as in QM/MM MD simulations. Electron density analysis was performed in the Multiwfn 3.7 software package [77].

## 4. Conclusions

To this date, the catalytic mechanism of LINE-1 ORF2p reverse transcriptase remains ambiguous due to high-resolution structural evidence of a single active-site Mg^2+^ cation, while generally DNA polymerases are supposed to employ the two-metal-ion mechanism. To resolve this discrepancy, we performed QM/MM simulations based on the high-resolution structure of the ternary complex of ORF2p RT domain with hybrid duplex and dNTP (PDB ID: 8C8J) that became available recently [9].

The computed biased QM/MM molecular dynamics trajectories reveal the concerted direct proton transfer from the O3′ to the Asp703 side chain and nucleophilic attack by DNA O3′, eliminating the need for metal coordination of the primer O3′. The computed free energy barrier is 15.1 ± 0.8 kcal/mol, and the products are stabilized by 7.8 ± 1.2 kcal/mol compared to the ES complex. We estimate the rate constant based on the simulation results at ~60 s^−1^. While no direct comparison can be made with the experimental kinetics of ORF2p RT, the computed rate constant matches with the known retrotransposon activity of MarathonRT and HIV-1 RT, benchmarking the simulations performed in this paper. Additionally, the analysis of the ES complex structure as well as constrained dynamics in the transition state region consistently reveal the type of reaction mechanism. Analysis of the Laplacian of the electron density calculated in the ES complex along the cleaving P-O_LG_ bond and in the plane of a phosphorus atom, P, and oxygen atoms, O_Nuc_ and O_LG_, shows that there is no electron density concentration area on the bond line, which is an indicator of the dissociative mechanism.

Our work hints that ORF2p RT mechanism diverges from the common one for the DNA polymerases. We demonstrate that Mg^2+^ cation has only a structural role and determines proper binding of the nucleotide for elongation of the DNA. The O3′ can be deprotonated with a reasonable energy barrier without polarization by another Mg^2+^ cation. In contrast to available theoretical studies on RT discussed in the Introduction, we were able to explicitly demonstrate the proton acceptor. The Asp703 is a key catalytic residue that acts as a close and efficient proton acceptor. It could be targeted to disrupt LINE-1 propagation that might assist therapies against LINE-1-mediated pathologies without cross-inhibiting DNA polymerases.

## Figures and Tables

**Table 1:** Decomposition of distributions of interatomic distances in the ES complex obtained in unconstrained QM/MM MD simulations. Mean value and standard deviation of each component of a linear combination of normal distributions is shown, and the values in parenthesis are weights.

d(P…O_LG_), Å	d(P…O_Nuc_), Å	d(P…O_LG_)-d(P…O_Nuc_), Å
1.653 ± 0.001 (1)	3.01 ± 0.02 (0.42)	−1.34 ± 0.02 (0.41)
	3.38 ± 0.02 (0.43)	−1.72 ± 0.02 (0.43)
	3.70 ± 0.05 (0.15)	−2.04 ± 0.05 (0.16)

## Data Availability

The original contributions presented in the study are included in the article/Supplementary Material, further inquiries can be directed to the corresponding author.

## Supplementary Materials

All QM/MM MD trajectories containing atoms of the QM part, reaction coordinate values at each MD frame, and coordinates of the entire system are available at Zenodo https://doi.org/10.5281/zenodo.17033960.
